# A Real-Time PCR based assay for determining parasite to host ratio and parasitaemia in the clinical samples of Bovine Theileriosis

**DOI:** 10.1038/s41598-018-33721-3

**Published:** 2018-10-18

**Authors:** Debabrata Dandasena, Vasundhra Bhandari, G. S. Sreenivasamurthy, Shweta Murthy, Sonti Roy, Vandna Bhanot, Jaspreet Singh Arora, Satparkash Singh, Paresh Sharma

**Affiliations:** 1 National Institute of Animal Biotechnology, Hyderabad, India; 2 Disease Investigation Laboratory, LUVAS, Ambala, Haryana India; 3 Department of Parasitology, College of Veterinary Science, Rajendra Nagar, Hyderabad India; 4 School of Animal Biotechnology, GADVASU, Punjab, India

## Abstract

*Theileria annulata* is an intracellular parasite that causes active and latent forms of bovine theileriosis. Diagnosis of the disease is primarily based on traditional methods such as microscopy, however, PCR based methods have proven to be superior in the absence of clear disease symptoms. However, diagnosis is difficult in cases of lower parasitaemia by conventional PCR. Hence, a rapid and sensitive method which can detect early infection and low parasite load is required. Therefore, we have developed an absolute quantification based real-time PCR (qPCR) assay. Reference standard curve using recombinant plasmids of a host (*hprt*) and a parasite gene (*tasp*) was constructed, and the assay was initially standardised using *in vitro T. annulata* cell lines. Further, 414 blood samples from suspected theileriosis cases were also evaluated using qPCR. The assay can estimate host to parasite ratios, calculate parasitaemia and treatment effectiveness in the clinical cases of theileriosis. In comparison with the conventional PCR results, 44 additional positive cases were found. Therefore, the assay holds importance in a clinical setting due to its ability to quantify the parasite load in clinical samples. It may be further used in distinguishing active and latent theileriosis infections and detection of drug resistance in the field.

## Introduction

Bovine theileriosis is caused by an apicomplexan parasite *Theileria spp*. which is an important tick-borne disease of livestock^1^. *Theileria annulata* and *Theileria parva* are the two economically important species responsible for livestock morbidity and mortality worldwide^2–4^. In India, *T. annulata* is the primary causative agent which hampers animal health and productivity^5,6^. The economic loss to the tune of $800 million has been reported due to infection caused by *T. annulata* in India^7^. It mainly infects cross-breed cattle, however, native breed cattle like water buffalo, and small ruminants are also known to be affected^5^. The prevalence of *T. annulata* from different parts of India has been reported from 3 to 41% with the help of microscopy and molecular tests^8,9^. The existing diagnostic tools include microscopy, PCR, and serological assays. The need of the hour is a sensitive, and reliable diagnostic tool which can perform timely detection along with an estimation of host-parasite ratio of clinical samples.

The life cycle of *T. annulata* is complex, the tick vector while feeding on cattle releases the sporozoites in the bloodstream, which later enters into the host leukocytes (monocytes or B-lymphocytes)^10,11^. Following host leukocyte invasion, *T. annulata* hijacks the host cell machinery and transforms the cells with a cancer-like phenotype^11^. *T. annulata* parasites multiply in synchrony along with the host cells and form schizonts which is the symptomatic stage of the disease. The transformed *T. annulata* infected bovine leukocyte cells can be cultured *in vitro* for an infinite time in the parasite-specific culture medium. Currently, an attenuated *T. annulata* schizont stage vaccine is available in India for controlling the disease^12,13^. The diagnosis of theileriosis heavily relies on the microscopy, where Giemsa stain is used to check for *Theileria* infected multinucleated host cells (Koch’s bodies) and the piroplasm stage in the blood smear^14^. Microscopy has certain drawbacks of being tedious, labour intensive, misleading (due to similar morphological features with other parasites like *Babesia*), and also require an expert technician^15,16^. However, the technique is not effective in early diagnosis due to low parasitaemia^16^.

Molecular diagnosis using conventional PCR based on several *T. annulata* specific genes, 18S rRNA, *T. annulata* merozoite surface protein (*tams*), *T. annulata* sporozoite surface protein (*tasp*) or Cytochrome III, and serological assays using TASP and TAMS antigens are still used for diagnostic purpose^16–20^. Recently, Real-time quantitative PCR based on the *18s rRNA* and *tams* genes have been used for detection of the *T. annulata* parasites^21–24^. The surfacing problem of drug resistance against the current anti-*Theileria* drug buparvaquone (BPQ) also presses the need for a better diagnostic tool in evaluating treatment response^22,25–27^. However, none of the current tools helps in identifying the host to parasite ratio, parasite burden, and chemotherapy response.

When compared to the other apicomplexan parasite like *Plasmodium falciparum*, which resides in RBC cell (nonnucleated), diagnosis of *Theileria* parasites poses challenge owing to their complex life cycle inside bovine leukocytes. The high sensitivity of the real-time PCR technique makes it an appropriate method for early disease diagnosis and parasite quantification^28,29^. In line with the current scenario, we have developed a qPCR assay which will help in identifying the parasite burden, host to parasite ratio and as well as treatment response in the field.

## Result

### Sensitivity, PCR Efficiency, and Standard curve analysis

A single copy gene specific (hypoxanthine phosphoribosyltransferase 1, *hprt*) to the host and the parasite (*Theileria* annulata surface protein, *tasp)* was used to quantify the host-parasite DNA^30^. The *hprt and* tasp were amplified using the gene-specific primers and cloned into the pBSK plasmid. The melting curve for the *tasp* and *hprt* showed a single peak at 78.72 °C and 84.57 °C, respectively for the reference plasmid DNA and the biological sample.

The PCR sensitivity was determined by serially diluted DNA from 10 ng to 1fg. Amplification was detected until the lowest DNA dilution of 1fg, and the Cq values ranged from 14.23 ± 0.28 (10 ng) to 30.94 ± 0.32 (1fg). The Gene Copy Number (GCN) of the two plasmids were calculated using the standard formula mentioned below in material and methods. The standard curve was plotted by serially diluting (1:10) the *hprt* and *tasp* plasmid constructs starting from the 10^6^ to 10 GCN. A Cq value was obtained for each dilution, and three replicates were used for each dilution. The average Cq values were plotted against each respective GCN dilutions for both the plasmid constructs (Table 1). The slope of the standard curve was found to be −3.04 for *hprt* plasmid and −3.12 for the *tasp* plasmid. The correlation coefficient (r^2^) for the PCR reaction was found to be 0.99 while the PCR efficiency was 112% and 109% for the *hprt* and the *tasp* plasmid, respectively. A negative template control and uninfected cattle DNA samples were run to check for any contamination during the PCR runs.

**Table 1:** Quantification of gene copies of *hprt* and *tasp* by qPCR.

Gene Copy Number	*hprt* (Cq ± SD)	*tasp* (Cq ± SD)
1.00E + 06	17.01 ± 0.12	18.80 ± 0.72
1.00E + 05	20.77 ± 0.57	21.27 ± 0.19
1.00E + 04	23.79 ± 0.06	25.05 ± 0.08
1.00E + 03	27.45 ± 0.33	28.65 ± 0.28
1.00E + 02	30.03 ± 0.10	31.75 ± 1.63
1.00E + 01	32.07 ± 0.25	33.59 ± 0.28

### Host-Parasite DNA Quantification

**Figure 1:**
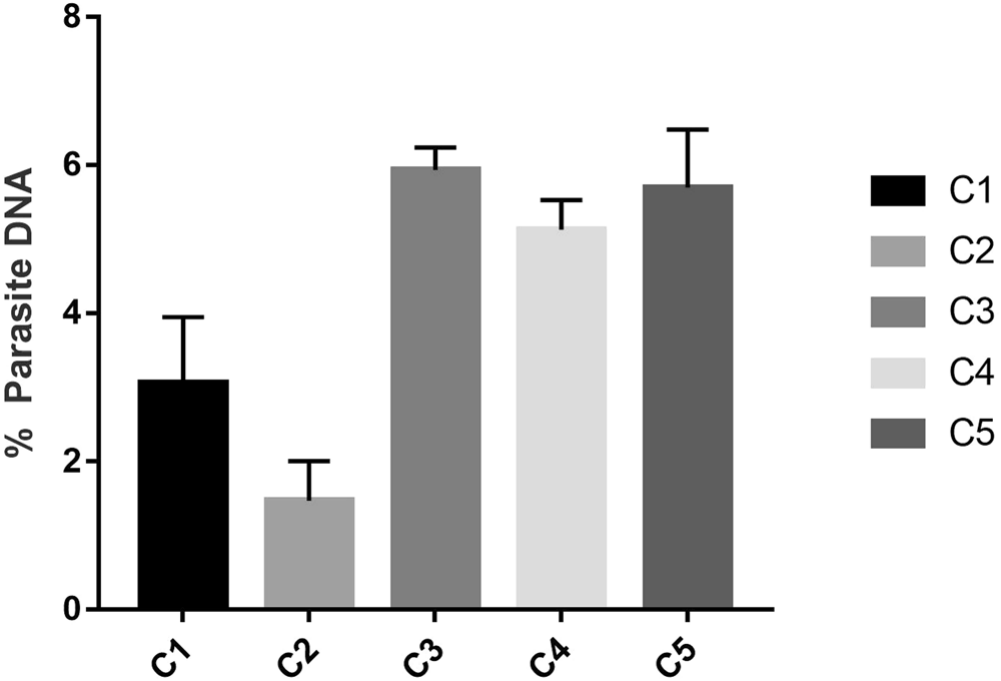
Parasite DNA percentage in the bovine cells infected with *Theileria annulata*.

The host-parasite DNA was quantified from the *in vitro T. annulata* infected host leukocytes cell lines. A qPCR was first performed using the *T. annulata* cell lines against the *hprt* and *tasp* genes. The Cq values obtained were used to calculate the GCN of the host and parasite DNA in the cell lines in reference to the standard curve plotted using the *hprt* and *tasp* plasmid constructs. The GCN of the host was divided by a factor of 2 owing to its diploid genome. The GCN values were further used to calculate the host and parasite DNA ratio using the equation 2. In the 5 cell lines used for DNA quantification, the parasite DNA (%) was found to be ranging from 1.47 ± 0.53% to 5.94 ± 0.30% (Fig. 1).

### Parasite load in the clinical cases of bovine theileriosis

**Figure 2:**
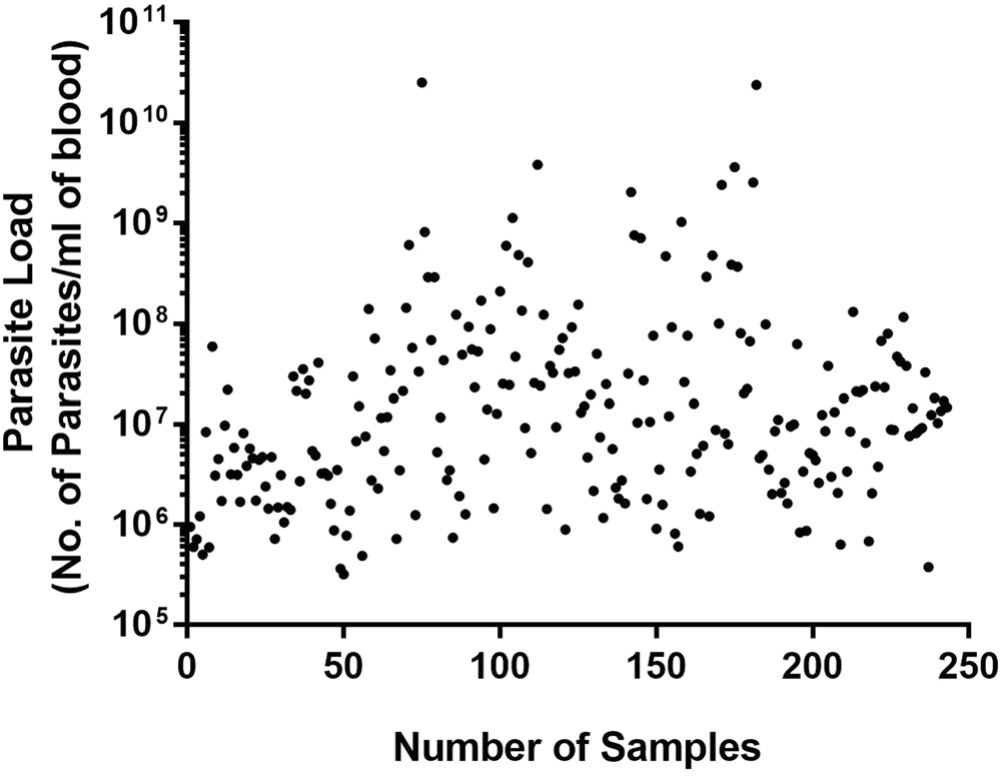
Scatter plot representing the parasitaemia (*T. annulata*/ml of blood) in blood samples of clinical cases. Parasite load was calculated for blood samples of cattle infected with *T. annulata*. Real-time PCR was carried out and the C_t_ value obtained was used for estimating the parasitaemia (*T. annulata*/ml).

The DNA was isolated from the whole blood collected from 414 cattle suspected for bovine theileriosis. PCR using 18S *rRNA* and *tasp* gene were done for all samples to check for *T. annulata* infection. Out of 414 samples, only 219 were PCR positive, however, when checked with real-time PCR 243 samples were found to be positive. Further, the C_q_ values obtained for each sample was used for identifying the parasite load in the clinical samples using equation mentioned in material and method. The parasite load ranged from 3.18E + 05 to 2.54E + 10 (Fig. 2). The samples in which C_q_ value ≥ 36.94 ± 0.25, i.e. the C_q_ value of negative template control (NTC) were considered as negative.

### Analysis of the Parasite DNA before and after drug treatment

**Figure 3:**
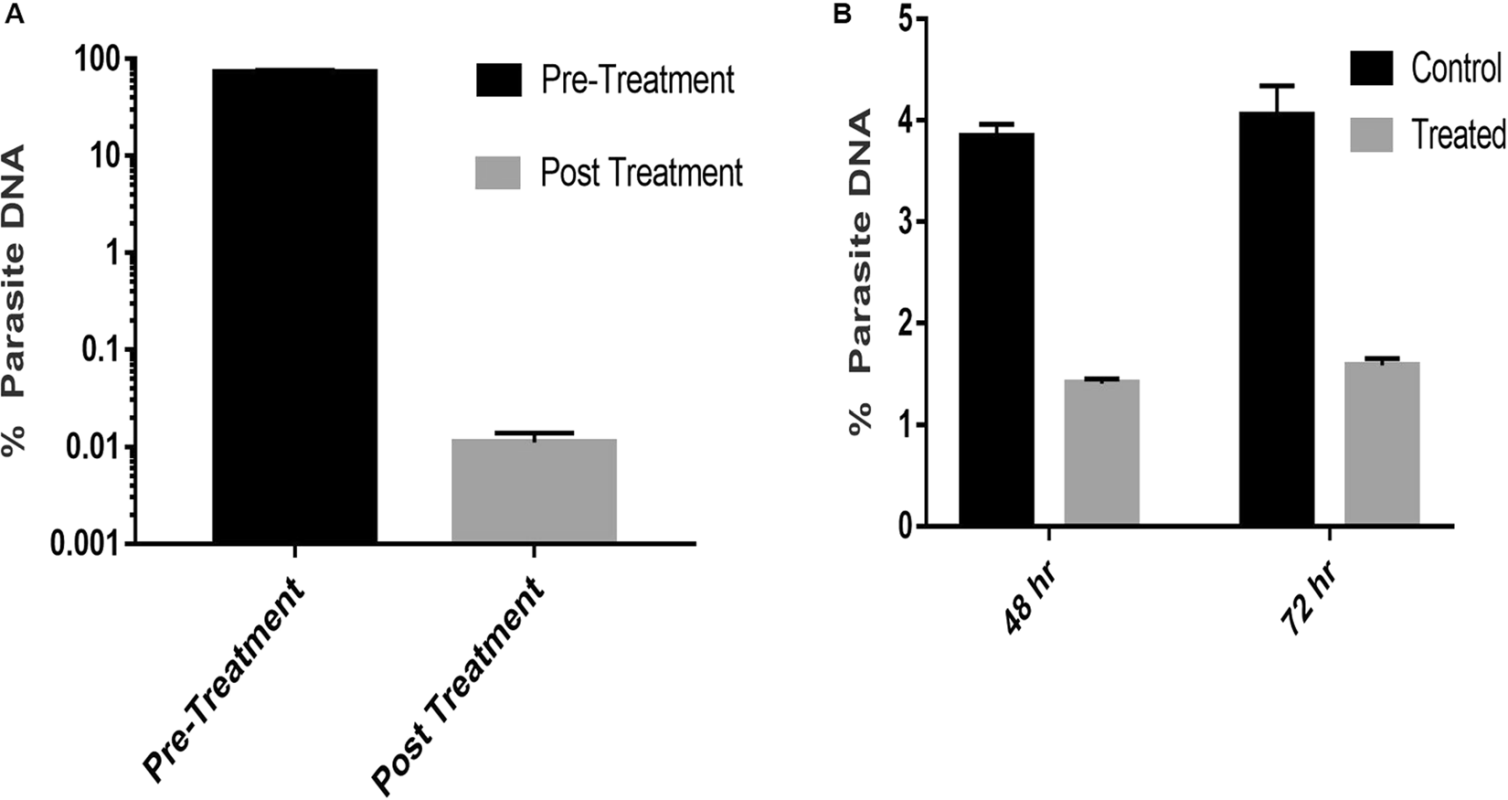
Reduction in % parasite DNA after Buparvaquone (BPQ) treatment. (**A**) A clinical case at pre-treatment and 10 days post-treatment with buparvaquone. (**B**) % Reduction of parasite DNA in *T. annulata* infected cell lines after treating with buparvaquone at 48 and 72 hours. The bar represents % parasite DNA ± SD.

To monitor the response of the chemotherapy, a blood sample was collected from infected cattle before and after 10 days after the treatment. Microscopy and, and real-time PCR analysis was done from the pre and post-treatment sample to determine the treatment efficacy. The real-time PCR analysis showed a reduction of parasite DNA from 72.54 ± 4.55% to 0.01 ± 0.003% after treatment, suggesting parasite clearance (Fig. 3A). Blood smears examination also revealed the absence of piroplasm in post-treatment as compared to pre-treatment blood sample. Similarly, real-time PCR was also performed on a DNA sample of cell lines treated and untreated with BPQ. DNA was isolated from the cell lines after 48 and 72 hrs of BPQ treatment to monitor the parasite growth. After, 48 hr the % parasite DNA was 3.84 ± 0.13% in control cell lines and got reduced to 1.41 ± 0.05% in treated cells (Fig. 3B). Similarly, after 72 hr, control cells exhibited 4.05 ± 0.29% parasite DNA, whereas, the treated cells had 1.55 ± 0.02% of parasitic DNA in them.

## Discussion

A quick, sensitive and specific diagnostic tool is a must for effective control of bovine theileriosis. qPCR has served as an efficient tool for detection and quantification of the parasites of various diseases. In the present study, we have developed a diagnostic assay which will help us in the diagnosis and quantification of *T. annulata* parasite and also monitor treatment effectiveness. Although the assay has limitations and could not differentiate between infected and vaccinated animals. Due to insufficient studies on the differences among vaccinated and *T. annulata* infected animals, until a date no molecules/gene have been identified which can differentiate between the two groups. The real-time PCR is based on the absolute quantification method using recombinant plasmids corresponding to single copy genes specific to the parasite, (*tasp)* and the host (*hprt)*.

In a previously published report, 18S rRNA based qPCR for detection and quantification of *T. annulata* was used, however, owing to its multi-copies in the single genome and sequence similarity among various *Theileria* and *Babesia* parasites, it may pose several disadvantages and limitations in testing clinical samples^30,31^. We have utilised parasite gene *tasp*, which is preferred over other determinants such as 18S rRNA, TAMS gene of *T. annulata* for diagnosis^23,31–33^. We have developed a qPCR assay, in which reference standard curves were generated for calculating host and parasite ratios in the clinical samples.

This approach has been utilised earlier on four *T. parva* cell lines, however, there is no such report on *T. annulata*^30^. In the cell lines analysed, we have found the % parasite DNA varied from 1.47% to 5.94%, which is in line with earlier studies on *T. parva* cell lines which showed it to be 0.9% to 3%. Less amount of % parasite DNA in the cell lines corresponds to the fact that parasite genome is quite smaller in comparison to bovine genome^30^. The advantage of calculating the host-parasite ratios will provide useful insights to understand host-pathogen biology and assist in various experimental approaches, e.g. use of sample with higher parasite load for performing whole genome sequencing of the parasite

Further, we have also performed qPCR on 414 bovine blood samples. 44 additional samples were diagnosed as infected by qPCR in comparison to conventional PCR due to presence of low parasitemia. The parasite load in the clinical samples was found to be ranging from 3.18E + 05 to 2.54E + 10 parasites/mL of blood. Ros-Garcia *et al*., 2012 reported the development of a real-time PCR assay for quantification of parasite load, however, it cannot calculate the host-parasite ratios^21^. However, with our assay, we can calculate both the host-parasite ratios and as well as parasitaemia in clinical samples. The assay also provides an additional benefit of quantifying parasite load in clinical samples, which would pave a way to distinguish between active and carrier animals. However, studies on more sample number and its correlation with the clinical profile will be required to establish the same.

BPQ was selected for treatment of theileriosis due to low toxicity and a long plasma half-life of 7 days^34^. However, a prepatent period of 10–13 days is observed from tick feeding on to cattle blood to onset of fever and clinical symptoms. If the diagnosis followed by the treatment is delayed, it results in animal death. Therefore, timely diagnosis, as well as monitoring treatment response are crucial for disease control and drug resistance. Earlier report has observed parasite clearance after 8–10 days of BPQ treatment^35^. Therefore, we monitored the parasite load in cattle before and after 10 days of treatment and observed a marked decrease in the parasitaemia indicating the treatment was effective. Similarly, the same approach was tested in *T. annulata* cell lines incubated with the BPQ for 48 to 72 hrs. In all the cases, we observed a decrease in the % parasite DNA, indicating parasite death.

This assay holds importance in calculating the ratio of the parasite DNA in the bovine host which will further help in answering various research questions. Further, it can lead to monitoring the decrease in parasite load in clinical cases to assess the animal clinical state and treatment effectiveness.

## Materials and Methods

### Sample Collection, DNA Isolation, and *Theileria* Specific PCR

Blood samples were collected from cattle belonging to different endemic regions of India. Samples were collected and preserved in EDTA by a trained veterinarian. A total of 414 blood samples were collected from suspected cases of theileriosis (showing clinical symptoms) from different states, Andhra Pradesh, Telangana, Punjab, and Haryana. Study design and reporting follow the Standards for the Reporting of Diagnostic accuracy (STARD-2015: http://www.stard-statement.org/) (S1 Flow Diagram).

Genomic DNA was isolated from 2 ml of blood samples using previously published DNA isolation protocol^1^. Quality and integrity of the DNA were checked using Nanodrop and by running the genomic DNA on 0.8% agarose gel. After the quality check DNA samples were stored at −80 °C until further experiments. *T. annulata* specific 18S *rRNA* and *tasp* gene primers were used for conventional and qPCR (Table 1).

The PCR conditions for the primers mentioned above are as follows:

18S *rRNA*: 95 °C for 3 min, followed by 35 cycles of 95 °C for 30 s, 61 °C for 30 s and 72 °C for 30 s, and a final extension of 3 min at 72 °C.

*tasp:* 95 °C for 3 min, followed by 35 cycles of 95 °C for 1 min, 55 °C for 1 min, 72 °C and 1 min and a final extension of 5 min at 72 °C 5 min.

gDNA from pre-established *in vitro* culture of *Theileria* infected bovine cell line was used as positive control, and no template control was used for ruling out any contamination.

### Cloning of the hprt and the tasp gene

The pBSK plasmid was used for cloning the genes. Briefly, the *hprt* and *tasp* genes were amplified from Bovine cells and *T. annulata* cell line respectively using the thermal cycles below:

*hprt*: 95 °C for 5 min, followed by 35 cycles of 95 °C for 1 min, 60 °C for 1 min, 72 °C for 1 min and a final extension of 72 °C for 5 min.

*tasp*: 95 °C for 3 min, followed by 35 cycles of 95 °C for 1 min, 55 °C for 1 min, 72 °C for 1 min and a a final extension of 72 °C for 5 min.

The primer sequences are given in Table 1. The amplified products were cloned into a pBSK plasmid using the TA cloning method as described earlier (https://www.thermofisher.com/in/en/home/life-science/cloning/ta-cloning-kits.html). Later pBSK was transformed into the Top10 cells competent *E. coli* cells, and cloned plasmids were selected by growing cells in an ampicillin medium. Cloned plasmids were confirmed by restriction digestion using XcmI and Sanger sequencing using gene-specific primers. The NCBI Nucleotide database was queried to confirm the gene sequences.

### qPCR SYBR Green Based Assay

Primers for use with intercalating dye-based qPCR were designed using Primer 3 software for *tasp* and *hprt* genes. The analysis was carried out on an Applied Biosystems 7500. Melting curve analysis was done for both the primers showing single and specific peaks. 6 Tenfold serial dilution (10^6^ to 10 GCN) of the pBSK-*hprt* and pBSK-*tasp* plasmids were used for generating the standard curve (Table 2).

**Table 2:** List of primers used in the study.

Gene	Sequence (5′-3′)	Product size (bp)
*hprt-for*	ATGGCGGCCCGCAGCCCCAGC	657
*hprt-rev*	TTAGGCTTTGTATTTTGCTTTTC	657
*tasp-for*	TTGCGAATGCGGTCCATTTC	1065
*tasp-rev*	CTGGCAGGGTGAGAACGTAA	1065
*18s-rrna-for*	ACGACTCCTTCAGCAC CTTG	125
*18s-rrna-rev*	AAATTAAGCCGCAGCTCCAC	125
*qhprt-for*	TGGACAGGACCGAACGGCT	115
*qhprt-rev*	TAATCCAACAGGTCGGCAAG	115
*qtasp-for*	ATAAGCGCCCGAAGGGTAAT	160
*qtasp-rev*	CCACCAGTCAAACGCTACAG	160

### *In vitro* culture of the *T. annulata* infected bovine lymphocyte cell line

*T. annulata* infected leukocyte culture were established by isolating the PBMCs from the clinically infected cattle^1^. Briefly, isolated PBMCs were cultured in the RPMI 1640 medium with 10% FBS and Pen/Strep (100 µg/ml) solution at 37 °C with 5% CO_2_. *T. annulata* infected bovine cells transform and continue to grow *in vitro* condition for an infinite time. *T. annulata* parasites inside the bovine cells were confirmed by PCR using *tasp* specific primers.

### Monitoring treatment response using qPCR assay

First, the efficacy of BPQ was checked on *T. annulata* cell line. 2.5 × 10^5^ cells/ml of *T. annulata* cell line was dispensed in a 6-well tissue culture plate. Further, BPQ was added at the concentration of 50 ng/ml to the test wells, and DNA was isolated from the wells after 48 and 72 hr of incubation. Cells without BPQ were treated as control, and gDNA was isolated. Similarly, the treatment response in a *T. annulata* infected cattle was also checked by collecting blood samples before and after 10 days of the BPQ treatment. All the experiments were done in triplicate. qPCR was done to check the parasitaemia in the cell line before and after treatment.

### GCN calculation

The gene copy of the plasmids was calculated using the below-mentioned equation^30^.

1 $$\frac{{\rm{GCN}}=6.02\times {10}^{23}\,({\rm{copy}}/{\rm{mol}}){\boldsymbol{\times }}{\rm{Amount}}\,{\rm{of}}\,{\rm{DNA}}\,({\rm{g}})}{\,{\rm{DNA}}\,{\rm{length}}\,({\rm{bp}}){\boldsymbol{\times }}{\rm{660}}\,({\rm{g}}/{\rm{mol}}/{\rm{bp}})}$$

In the equation,

$${\rm{DNA}}\,{\rm{length}}={\rm{length}}\,{\rm{of}}\,{\rm{plasmid}}\,(2958\,{\rm{bp}})+{\rm{length}}\,{\rm{of}}\,{\rm{insert}}\,({\rm{tasp}}=1065\,{\rm{bp}},\,{\rm{hprt}}=657\,{\rm{bp}}).$$

$${\rm{Amount}}\,{\rm{of}}\,{\rm{DNA}}={\rm{Plasmid}}\,{\rm{concentration}}$$

The cloned *tasp* and *hprt* gene in a pBSK cloning vector (pBSK-tasp & pBSK-hprt) were 10 fold serially diluted up to 6 dilutions ranging from 10^6^ to 10 copy/µl. The standard curve was plotted as copy number vs. C_q_ value for each of the dilutions in triplicates for pBSK-*tasp* and pBSK-*hprt* respectively.

### Estimation of Host-Parasite DNA ratio

The host to parasite DNA ratio was calculated using equation 2 as mentioned below. As the bovine cells are diploid, the number of bovine cells were considered to be half the GCN of the *hprt* gene. As the parasite is in the haploid state while surviving inside host cells, the number of parasite cells is considered equivalent to GCN of the *tasp* gene^30^. The GCN was then used to calculate the amount of host and parasite DNA in the tested sample.

$$\begin{array}{c}{\rm{Host}}=({\rm{GCN}}/2)\times {\rm{Mass}}\,{\rm{of}}\,{\rm{Bovine}}\,{\rm{genome}}\,(0.0033066)\,{\rm{ng}}\\ \,\mathrm{Parasite}:\,{\rm{GCN}}\times 0.00000914826\,{\rm{ng}}\,({\rm{Mass}}\,{\rm{of}}\,{\rm{parasite}}\,{\rm{genome}})\end{array}$$

2 $${\rm{Ratio}}=[{\rm{Parasite}}\,{\rm{DNA}}/{\rm{Total}}\,{\rm{DNA}}\,({\rm{Host}}+{\rm{Parasite}})]{\boldsymbol{\times }}100$$

### Parasite Load Calculation

The parasitaemia in each sample was calculated as described earlier^21^. Briefly, the estimated parasite gene copy number (Q) obtained from the above-described method was used in the below-mentioned equation to calculate the *T. annulata* cells per ml of blood

$${\rm{Parasitaemia}}\,({\rm{P}})={\rm{Q}}\times ({{\rm{V}}}_{{\rm{B}}}/{{\rm{V}}}_{{\rm{EX}}})\times ({{\rm{V}}}_{{\rm{EL}}}/{{\rm{V}}}_{{\rm{T}}})\times (1/{\rm{CN}})$$

where V_B_ = Volume of blood, 1000 ul

V_EX_ = sample volume extracted, 2000 ul

V_EL_ = Elute volume, 100 ul

V_T_ = Template used for PCR, 1 ul

CN = Gene copy number (2 copies per genome)

### Ethical approval and informed consent

Oral consent was taken from the farm owners before drawing blood from animals. There is no specific law in India which requires permission from the ethics committee for collecting less than 5 ml of blood. Further, blood samples were collected by professional veterinarians.

## Data Availability

The datasets generated during and/or analysed during the current study are available from the corresponding author on reasonable request.
